# Interaction between Perceived Autonomy Support and Sports Engagement of Athletes: A Cross Lag Regression Analysis

**DOI:** 10.5114/jhk/203026

**Published:** 2025-09-23

**Authors:** Zhao Da-liang, Zhou Yu

**Affiliations:** 1 Department of Sport and Health, Guangzhou Sport University, Guangzhou, China.; 2 Guangdong Provincial Key Laboratory of Human Sports Performance Science, Guangzhou, China.

**Keywords:** reciprocal influence, coaching style, athlete-coach interaction

## Abstract

To examine the relationship between perceived autonomy support and sports engagement among athletes, this study conducted a 6-month follow-up with 79 provincial team athletes, assessed over three time periods. The results were analyzed using latent variable growth models and cross-lagged regression analysis. The findings indicated that: (1) both perceived autonomy support and sports engagement exhibited a linear decline across the three measurement periods; (2) perceived autonomy support significantly predicted subsequent sports engagement, and sports engagement, in turn, significantly predicted later perceived autonomy support. However, in terms of stability, perceived autonomy support was a more consistent predictor of sports engagement. In summary, these findings reveal the dynamic interaction between athletes' perceived autonomy support and sports engagement, highlighting the crucial role of coaches' autonomy support in shaping athletes' engagement, as well as the reciprocal influence of athletes' engagement on the coaching style.

## Introduction

Just as parenting styles play a significant role in shaping children's psychological experiences within the family environment (Vega-Díaz and González-García, 2025; Yang and Zhao, 2020), coaches play a pivotal role in the development of athletes' physical and psychological functioning within sports settings (Adie et al., 2008). Specifically, coaching styles have a profound impact on athletes' psychological experiences (Adie et al., 2008). Among these styles, autonomy-supportive coaching has attracted considerable attention from researchers. Autonomy-supportive coaching refers to a style in which the coach actively nurtures the athlete’s sense of autonomy by providing opportunities for choice, promoting a sense of volition, and fostering self-endorsement (Prusak et al., 2004). Therefore, it is essential to investigate the effects of perceived autonomy support on athletes (Huh and Choi, 2017).

Based on Deci and Ryan's (1980) self-determination theory, researchers have demonstrated that autonomy support is linked to positive cognitive, affective, and behavioral outcomes in athletes (Mageau and Vallerand, 2003; Reinboth et al., 2004; Stebbings et al., 2016). For instance, coaches' autonomy-supportive coaching styles have been shown to significantly enhance athletes' sport engagement during training (Balk et al., 2019; Cheon et al., 2015). Sport engagement is a stable, positive cognitive-emotional experience characterized by enthusiasm, confidence, dedication, and vitality (Lonsdale et al., 2007). According to Lonsdale et al. (2007), sport engagement is crucial for elite athletes due to the significant time and energy required to excel in their sport. Furthermore, sport engagement has been associated with positive outcomes such as experiencing a state of flow in exercise contexts (Hodge et al., 2009). In the field of sports, a large number of studies have shown that autonomy support is related to sports engagement (Curran et al., 2014; Reynders et al., 2019).

Several studies have examined the predictive role of perceived autonomy support on athletes' sport engagement (Haerens et al., 2018; O'Neil et al., 2020). However, while these studies have highlighted the important role of autonomy support in sport engagement, most of them have primarily used cross-sectional research methods and have not examined the reverse effect of athletes' sport engagement on perceived autonomy support. This gap may arise from the fact that most studies have employed a cross-sectional research methodology to analyze the effect of perceived autonomy support on sport engagement using structural equation modeling (Bormann et al., 2016; Cronin et al., 2015), which represents a unidirectional approach that only investigates the effect of perceived autonomy support on sport engagement, without considering the reverse influence of sport engagement on perceived autonomy support. Whether a bi-directional relationship between autonomy support and sport engagement exists in sport contexts has yet to be determined. Establishing such a relationship would enhance the understanding of movement patterns in sporting situations and play a significant role in athletes’ development.

We conducted a six-month follow-up study with three assessment points. The results were analyzed using latent variable growth modeling and cross-lagged modeling. This helped us explore the causal relationship between perceived autonomy support and sport engagement. First, perceived autonomy support and sport engagement were measured at three different timepoints: Time 1, Time 2, and Time 3. We analyzed the trends of both variables. This analysis contributed to a better understanding of their relationship.

Second, we tested the lead-lag relationship between perceived autonomy support and sport engagement using cross-lagged regression modeling. Previous research has shown that autonomy-supportive coaching increases athletes' sport engagement (Balk et al., 2019; Cheon et al., 2015). In education, student engagement also affects perceived autonomy support (Li et al., 2022). Based on these findings, we hypothesized that autonomy support and sport engagement would influence each other.

## Methods

### Participants

Participants of this study were athletes from the Guangdong Provincial Team. A total of 79 individuals were included: 42 males and 37 females. The average age was M = 17.72, SD = 3.73 years, and average training experience was M = 8.58, SD = 3.84 years. The sports included weightlifting, swimming, gymnastics, table tennis, volleyball, and martial arts.

Tracking high-level athletes from the provincial team was challenging, resulting in a significant loss of samples. To achieve a moderate effect size (f^2^ = 0.15, R^2^ = 0.13) with a power of 0.80 and the level of significance of 0.05 (Cohen, 1988), a minimum of 68 subjects was required. The final sample size for this study was 79. All procedures were approved by the ethics committee of the Guangzhou Sport University, Guangzhou, China (protocol code: 7220180231; approval date: 20 May 2021), and informed consent was obtained from all subjects or their parents.

### Measures

#### Sport Engagement

The Athlete Engagement Questionnaire (AEQ) (Curran et al., 2015; Lonsdale et al., 2007) is used to assess athletes' levels of engagement during training. The questionnaire comprises four subscales: confidence (e.g., “I am confident in my ability”), dedication (e.g., “I am committed to achieving my goal”), and enthusiasm (e.g., “I am very enthusiastic”). Responses are rated on a 5-point scale, ranging from 1 (almost never) to 5 (almost always). Previous studies have demonstrated the AEQ's effectiveness and reliability, confirming its factor structure and internal consistency through confirmatory factor analysis (CFA) (Cronbach's alpha ≥ 0.80; Draugelis et al., 2014). The internal consistency score in this study was 0.887. Given the high internal consistency, we used the total score of sport engagement, calculated as the sum of 16 items, as a manifest variable in our analysis.

#### Perceived Autonomy Support

The Perceived Autonomy Support Scale (Wilson et al., 2009) is used to evaluate athletes' perceptions of their coaches' autonomy support. The scale is unidimensional and consists of 6 items, such as “I feel my coach gives me the power to choose”, rated using a 7-point Likert rating scale. The Perceived Autonomy Support Scale has demonstrated good psychometric properties in sports contexts, with a Cronbach's alpha of 0.88. In this study, the internal consistency score was 0.858. Given the relatively high internal consistency coefficient, we used the total autonomy support score, calculated as a manifest variable in our analysis.

### Design and Procedures

Three measurements of sport engagement and perceived autonomy support were administered to athletes in the provincial team over a six-month period, with assessments at the beginning of the season, in the mid-season, and at the end of the season, spaced three months apart. The first measurement (T1) included a total of 338 valid participants. Of these, 192 also participated in the second measurement (T2). The third measurement (T3) included 79 valid participants, all of whom had also taken part in both the first and second measurements. This final group consisted of 42 males and 37 females.

### Statistical Analysis

First, descriptive statistics and correlation analyses were conducted for perceived autonomy support and sport engagement. Next, unconditional latent variable growth modeling was applied to each of the three measures of perceived autonomy support and sport engagement, with intercepts representing initial levels and slopes indicating changes, to examine the developmental trajectories of athletes' perceived autonomy support and sport engagement. We then performed cross-lagged regression analysis to further establish the temporal sequence and the overall causal direction between perceived autonomy support and sport engagement.

Data were analyzed using SPSS 22.0 and Mplus 8.0. The latent variable growth model and the cross-lagged regression model were estimated using a robust maximum likelihood estimator (MLR), as the K-S test results indicated that the sport engagement and autonomy support data were somewhat skewed across the three waves of observations. MLR has been shown to handle non-normal data more effectively than other methods (Bandalos, 2014). Given the degree of deviation from normality and the proportion of missing data (Dong and Peng, 2013), we retained only the sample data that completed all three assessments for analysis. Based on Hu and Bentler's (1999) recommendations, the model fit was evaluated using χ^2^, df, CFI (>0.90), GFI (> 0.90), TLI (>0.90), RMSEA (< 0.08), and SRMR (< 0.08).

## Results

### Common Method Bias

To assess the potential impact of common method bias on the three measurements, we conducted an exploratory factor analysis (EFA). All variables were included in the EFA, and the results of the unrotated factor solution were examined. We specifically tested the hypothesis of a “one-factor” model by restricting the number of factors to one. The fit for the one-factor model was poor, χ^2^/df = 2.680, CFI = 0.424, TLI = 0.406, RMSEA = 0.146, SRMR = 0.113, indicating that common method bias was not a significant concern.

### Descriptive Statistics and Correlation Analysis

Table 1 presents the means, standard deviations, and the correlation matrix for the three measurements of perceived autonomy support and sport engagement. From T1 to T3, both perceived autonomy support and sport engagement among athletes demonstrated a gradual decline. Additionally, the three measurements of perceived autonomy support and sport engagement were significantly positively correlated (*rs* = 0.219 to 0.972, *p* ≤ 0.05).

**Table 1:** Means, standard deviations and the correlation matrix of variables.

Variables	M				Correlation					
		SD	Skewness	Kurtosis	1	2	3	4	5	6
T1Engagement	52.987	9.448	−0.053	−0207	1					
T2Engagement	45.063	8.331	−0.053	−0459	0.972^**^	1				
T3Engagement	36.215	7.068	−0.040	−0.673	0.845^**^	0.944^**^	1			
T1Autonomy Support	27.911	4.641	−0.052	0.231	0.651^**^	0.751^**^	0.815^**^	1		
T2Autonomy Support	24.068	5.832	0.106	−0.639	0.565^**^	0.741^**^	0.914^**^	0.784^**^	1	
T3Autonomy Support	20.519	6.101	0.266	−0.624	0.219^*^	0.425^**^	0.662^**^	0.593^**^	0.869^**^	1

Note:* p ≤ 0.05; ** p ≤ 0.01; *** p ≤ 0.001

### Sport Engagement

**Figure 1:**
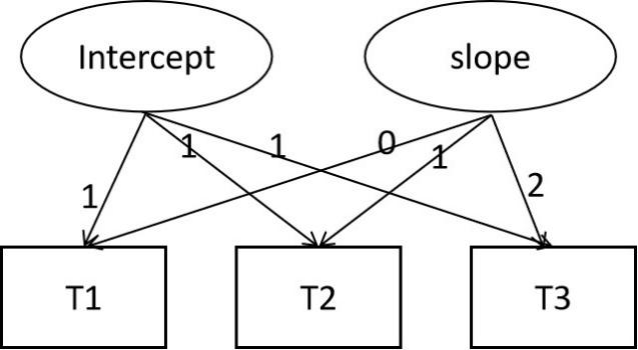
A linear unconditional latent variable growth model (T1 represents the first measurement time, T2 represents the second measurement time, T3 represents the third measurement time, all the same below).

We constructed a linear unconditional latent variable growth model (Figure 1) to examine the trend in sport engagement. The fit indices for sport engagement are presented in Table 2, and the unconditional model demonstrated a good fit to the data. The statistical results of the model are shown in Table 3.

**Table 2:** Fit indices of the linear unconditional latent variable growth model for perceived autonomy support and sport engagement models.

	χ^2^/*df*	*p*	CFI	TLI	RMSEA	SRMR
Engagement	0.164	0.686	1.000	1.004	0.000	0.001
Autonomy Support	0.053	0.817	1.000	1.015	0.000	0.006

**Table 3:** Parameter estimation results of latent variable growth model for perceived autonomy support and sport engagement.

Model	Coefficients		Variances		r
	intercept	slope	slope	Slope	
Engagement	5.302^**^	−3.118^**^	103.272^**^	8.027^**^	−0.746^**^
Autonomy Support	5.479^**^	−2.253^**^	40.583^**^	11.832^**^	−0.399^**^

In the linear unconditional growth model, the intercept, representing the initial level of athletes' engagement, was 5.302 (SE = 0.438, *p* < 0.001), which was significantly greater than 0. Sport engagement showed a significant decline over the course of the three measurements (slope = −3.118, SE = 0.280, *p* < 0.001). Additionally, the variance of the intercept (*σ*^2^ = 103.207, SE = 16.460, *p* < 0.001) was significant, as was the variance of the slope (*σ*^2^ = 8.203, SE = 1.319, *p* < 0.001). This indicates that there was substantial inter-individual variability in the initial level of sport engagement, and that changes in sport engagement over time varied systematically across individuals.

### Perceived Autonomy Support

In the linear unconditional latent variable growth model, the fit indices for perceived autonomy support are presented in Table 2, and the unconditional model demonstrated a good fit to the data. The statistical results of the model are shown in Table 3.

In the linear unconditional growth model, the intercept, representing the initial level of autonomy support perceived by the athletes, was 5.479 (SE = 0.509, *p* < 0.001), which was significantly greater than 0. Perceived autonomy support showed a significant decline over the course of the three measurements (slope = −2.253, SE = 0.223, *p* < 0.001). Additionally, the variance of the intercept (*σ*^2^ = 40.583, SE = 7.346, *p* < 0.001) was significantly greater than 0, as was the variance of the slope (*σ*^2^ = 11.832, SE = 2.148, *p* < 0.001). These results suggest that there were significant interindividual differences in the initial levels of autonomy support perceived by athletes, and that changes in perceived autonomy support over time varied systematically across individuals.

### The Lead-Lag Relationship between Perceived Autonomy Support and Sport Engagement

Cross-lagged regression analyses were performed to examine the lead-lag relationship between perceived autonomy support and sport engagement, providing stronger evidence for causal direction. Latent variable growth modeling enhances our understanding of the dynamic properties of individual variables. Cross-lagged regression controls for the autoregressive effects of each variable by setting stability coefficients, making it one of the most effective methods for testing the 'pure' directional effects between variables (Preacher, 2015). This approach allows us to evaluate the extent to which one variable predicts the other.

**Figure 2:**
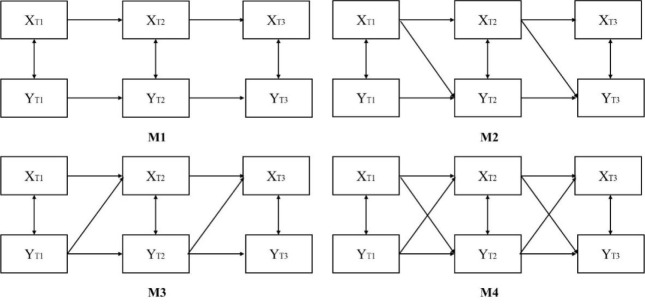
Cross lagged regression model diagram (X represents sport engagement, Y represents autonomy support).

A growing number of researchers suggest that to draw more robust conclusions in causal inference, combining multiple methods for sensitivity analyses should be considered (Curran and Bollen, 2001; De Stavola et al., 2006; Pakpahan et al., 2017). When using cross-lagged regression to explore causal relationships, four models need to be tested: (1) the baseline model (M1), which includes only autoregressive effects (Figure 2, M1); (2) the conceptualization model (M2), which adds the path from variable X to variable Y in M1 (Figure 2, M2); (3) the competing model (M3), which adds the path from variable Y to variable X in M1 (Figure 2, M3); and (4) the full model (M4), which includes all paths from M1, M2, and M3 (Figure 2, M4).

Table 4 shows the fitting indices of the four models. According to Table 4, the model fitting results of M4 were better than those of M1, M2, and M3.

**Table 4:** Model fitting index.

Models	χ^2^	*df*	CFI	GFI	SRMR	RMSEA
M1	323.360	8	0.724	0.518	0.365	0.706
M2	282.778	6	0.758	0.436	0.186	0.764
M3	55.763	6	0.957	0.899	0.070	0.324
M4	9.848	4	0.995	0.982	0.001	0.000

**Figure 3:**
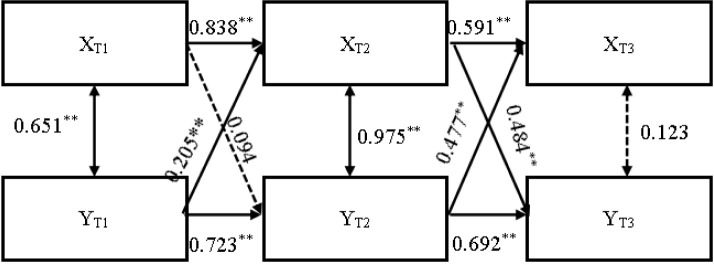
Cross lagged regression model.

The final model of perceived autonomy support and sport engagement is presented in Figure 3. Sport engagement at T1 (XT1, β = 0.838, SE = 0.025, *p* < 0.001) and perceived autonomy support at T1 (YT1, β = 0.205, SE = 0.030, *p* < 0.001) significantly predicted sport engagement at T2 (XT2). Similarly, sport engagement at T2 (XT2, β = 0.591, SE = 0.024, *p* < 0.001) and perceived autonomy support at T2 (YT2, β = 0.477, SE = 0.025, *p* < 0.001) significantly predicted sport engagement at T3 (XT3).

Perceived autonomy support at T1 (YT1, β = 0.723, SE = 0.077, *p* < 0.001) significantly predicted perceived autonomy support at T2 (YT2), but sport engagement at T1 (XT1, β = 0.094, SE = 0.091, *p* = 0.302) did not significantly predict perceived autonomy support at T2 (YT2). At T2, both sport engagement (XT2, β = 0.692, SE = 0.057, *p* < 0.001) and perceived autonomy support (YT2, β = 0.484, SE = 0.074, *p* < 0.001) significantly predicted perceived autonomy support at T3 (YT3).

## Discussion

We employed a linear unconditional growth model and cross-lagged regression analysis to examine the developmental trajectories of perceived autonomy support and sport engagement, as well as the causal relationship between the two variables. The results indicated that: (1) both sport engagement and perceived autonomy support exhibited a linear decline across the three measurement periods; and (2) cross-lagged regression analyses revealed a dynamic interaction between sport engagement and perceived autonomy support.

### Linear Changes in Perceived Autonomy Support and Sport Engagement

The results of the unconditional latent variable growth model indicate that athletes' levels of sport engagement and perceived autonomy support exhibit a significant linear decline (*p* < 0.001) over the three measurement periods, confirming Hypothesis 1. This decline in sport engagement and perceived autonomy support may be attributed to the unique characteristics of the athletic environment. Unlike non-sport settings, such as educational environments, a coach’s focus on an athlete often depends on the evaluation of competition or training outcomes. In these contexts, coaches are not only evaluated on their own merit, but also on the performance of their athletes (Cunningham and Dixon, 2003), which increases pressure on them. As competition approaches, this pressure can lead to a shift toward a less autonomy-supportive coaching style, which is then conveyed to the athletes (Rocchi and Pelletier, 2017; Stebbings et al., 2012). According to Wang (2024), the attentional network is affected by emotions. Consequently, the combination of prolonged stress and sustained high-intensity training results in consistently lower levels of athlete engagement and perceived autonomy support.

This dynamic change provides a basis for analyzing the causal relationship between the two variables. We should consider these dynamics when studying the relationships between explanatory variables. This will help us better understand and predict interactions in complex systems.

#### Dynamic Interaction between Perceived Autonomy Support and Sport Engagement

By comparing the models, we found that Model 4 had the best fit (Table 4), with perceived autonomy support at T1 and T2 significantly predicting sport engagement at the next time point. However, the prediction of perceived autonomy support by sport engagement was inconsistent. While sport engagement at T2 significantly predicted perceived autonomy support at T3, the effect of sport engagement at T1 on perceived autonomy support at T2 was not significant.

These findings indicate a reciprocal effect between sport engagement and perceived autonomy support over time. At the start of the season, this interaction begins with the environment (perceived autonomy support) influencing the athletes (sport engagement). Over time, athletes also begin to influence the environment, validating the hypothesis.

The mutual influence between perceived autonomy support and sport engagement can be explained as follows: first, when coaches adopt an autonomy-supportive style, they create a more motivating atmosphere, which promotes athletes' engagement. Second, when athletes show confidence, enthusiasm, energy, and focus during training, they exhibit greater initiative and achievement motivation (Deci and Ryan, 1980). In response, coaches may provide more choices, guidance, and positive feedback, further enhancing sport engagement.

Notably, the effect of autonomy support on sport engagement was stable, while the reverse effect was less consistent. This suggests that in high-pressure sports settings, athletes' sport engagement does not always influence coaches' autonomy-supportive behavior, which may vary based on time stages. However, coaches' autonomy support consistently affects athletes' engagement levels.

## Practical Implications

The results showed that perceived autonomy support had a more stable effect on sport engagement than the reverse. This suggests that coaches play a more dominant role in the coach-athlete relationship. This finding is consistent with self-determination theory. According to this theory, human behavior is largely shaped by how well the environment supports their autonomy, competence, and relatedness needs (Deci and Ryan, 1980, 1985).

In the future, interventions and improved coaching methods can be implemented to create a positive motivational climate for athletes. It is important to enhance coaches' training. This will help them better understand their dominant role in shaping the environment. By optimizing their coaching methods, coaches can promote positive experiences for athletes and improve the quality of training.

The results also highlighted the importance of athletes' self-regulation. The athletes' level of sport engagement influenced their perceived autonomy support. Previous interventions on coaching methods have mainly focused on coaches (Bartholomew et al., 2009; Raabe et al., 2019; Reynders et al., 2019). However, no studies have looked at interventions involving both coaches and athletes simultaneously. Based on the findings, improving both athletes' sport engagement and coaching styles may be more effective. This approach could enhance the quality of training and improve athletes' well-being.

Additionally, the study offers hope to athletes struggling in controlled environments. Even in stable, controlled settings, athletes can actively interact with their environment and increase their influence through their own efforts. Athletes are not merely passive recipients of their coaches' influence. Sport engagement can change how they perceive the autonomy and support they receive from their coaches. In fact, sport engagement can help create an environment that better supports their development.

As Albert Bandura stated, “Human beings are not only capable of transcending the dictates of their immediate environment, but are uniquely equipped to shape the circumstances and paths of their lives. People are not only products of their life circumstances, but also contributors to them” (Bandura, 2006).

## Limitations

Although the results established a causal relationship between perceived autonomy support and sport engagement, there are still some limitations. First, the sample size was relatively small. Due to the limited availability of high-level athletes, only 79 participants completed all three tests. While this sample size met the research requirements, future studies should consider using larger samples. Increasing the sample size and extending the tracking period could help capture the development trends and relationships between variables more comprehensively. This would allow for a more stable understanding of the long-term effects of coaching styles and sport engagement over time.

Secondly, the dimensions of the test variables in this study may be somewhat limited. This limitation is reflected in two aspects. First, coaching styles likely have multidimensional structures (Appleton et al., 2016) and are not limited to autonomy support. This study did not explore the relationship between other coaching styles and sport engagement. Second, in the sports environment, variables such as happiness and motivation are also important psychological factors, in addition to sport engagement. Future research could further investigate the dynamic interaction between coaching styles and these psychological variables.

## Conclusions

First, sport engagement and perceived autonomy support dynamically changed in competitive sport settings, both showing a decreasing trend over a six-month period.

Second, the relationship between perceived autonomy support and sport engagement was reciprocal. The effect of autonomy support on sport engagement was more stable than the effect of sport engagement on perceived autonomy support.
